# Long-term surviving cancer patients as a source of therapeutic TCR

**DOI:** 10.1007/s00262-019-02468-9

**Published:** 2020-01-08

**Authors:** Else Marit Inderberg, Sébastien Wälchli

**Affiliations:** Department of Cellular Therapy, Department of Oncology, Oslo University Hospital, The Norwegian Radium Hospital, 0379 Oslo, Norway

**Keywords:** T cell receptor, Adoptive cell therapy, Vaccination, CD4 T cells, Cancer immunotherapy, PIVAC 19

## Abstract

We have established a platform for the isolation of tumour-specific TCR from T cells of patients who experienced clinical benefit from cancer vaccination. In this review we will present the rationale behind this strategy and discuss the advantages of working with “natural” wild type TCRs. Indeed, the general trend in the field has been to use various modifications to enhance the affinity of such therapeutic TCRs. This was done to obtain stronger T cell responses, often at the cost of safety. We further describe antigen targets and recent in vitro and in vivo results obtained to validate them. We finally discuss the use of MHC class II-restricted TCR in immunotherapy. Typically cellular anti-tumour immune responses have been attributed to CD8 T cells; however, we isolated mainly CD4 T cells. Importantly, these MHC class II-restricted TCRs have the potential to induce broad, long lasting immune responses that enable cancer control. The use of CD4 T cell-derived TCRs for adoptive immunotherapy has so far been limited and we will here discuss their therapeutic potential.

## Introduction

### Adoptive cell therapy

Adoptive cell therapy (ACT) has been greatly enhanced when the injected T cells were genetically modified to express specific antigen receptors. In cancer therapy, two types have emerged, the natural T cell receptor (TCR) and the artificial Chimeric Antigen Receptor (CAR) [1]. The latter is a fusion of different protein domains, normally consisting of an antibody recognition domain on its external part which will be targeted towards a surface antigen. This extracellular domain is, through a transmembrane domain, connected to a series of signalling modules derived from the TCR signalling machinery. Despite the recent success and approval of CAR therapy for haematological malignancies [2] treatment of solid tumours still represents a challenge [3].

TCR therapy may have certain advantages over CARs for attacking solid tumours such as the number of targets available. Indeed, TCRs could in principle recognize any protein expressed by a cell, because all proteins are processed which in turn generates peptides that will be loaded on Major histocompatibility complex (MHC) class I and II molecules, whereas CAR recognition is restricted to surface molecules, limiting the tumour-specific pool of target antigens available. Furthermore, TCR recognition is highly subtle, and a single amino acid change on the target peptide can trigger the effector function of the TCR. Hence, peptides presented on MHC molecules can be derived from mutated proteins, rendering the recognition cancer-specific, an attribute that -although not impossible- is challenging to achieve with an antibody. Another argument in favour of TCR is that these molecules can be more sensitive to lower antigen densities on the target cell surface compared to CAR [4]. Direct comparisons of TCR and CAR are difficult because of differences in the ligands recognized and antigen. When Harris et al. [5] compared TCR αβ heterodimers and single-chain TCRs coupled to CAR signalling tails, both recognizing the same peptide-MHC (pMHC) complexes, they demonstrated that CARs were much less sensitive than TCR to lower pMHC densities, but induced higher cytokine secretion. Finally, although the cytokine response generated by classical CAR molecules upon stimulation is often far more intense than what is observed with TCRs, recent data demonstrated that lower CAR-derived T-cell stimulation might improve clinical outcome [6, 7]. In this perspective TCR-modified T cells can be seen as “soft” living drugs, yet able to generate serious side effects [8, 9] compared to CAR-T and, if well calibrated, they should represent the solution of choice for solid tumour-based ACT.

### Isolating TCRs from vaccinated patients

**Fig. 1:**
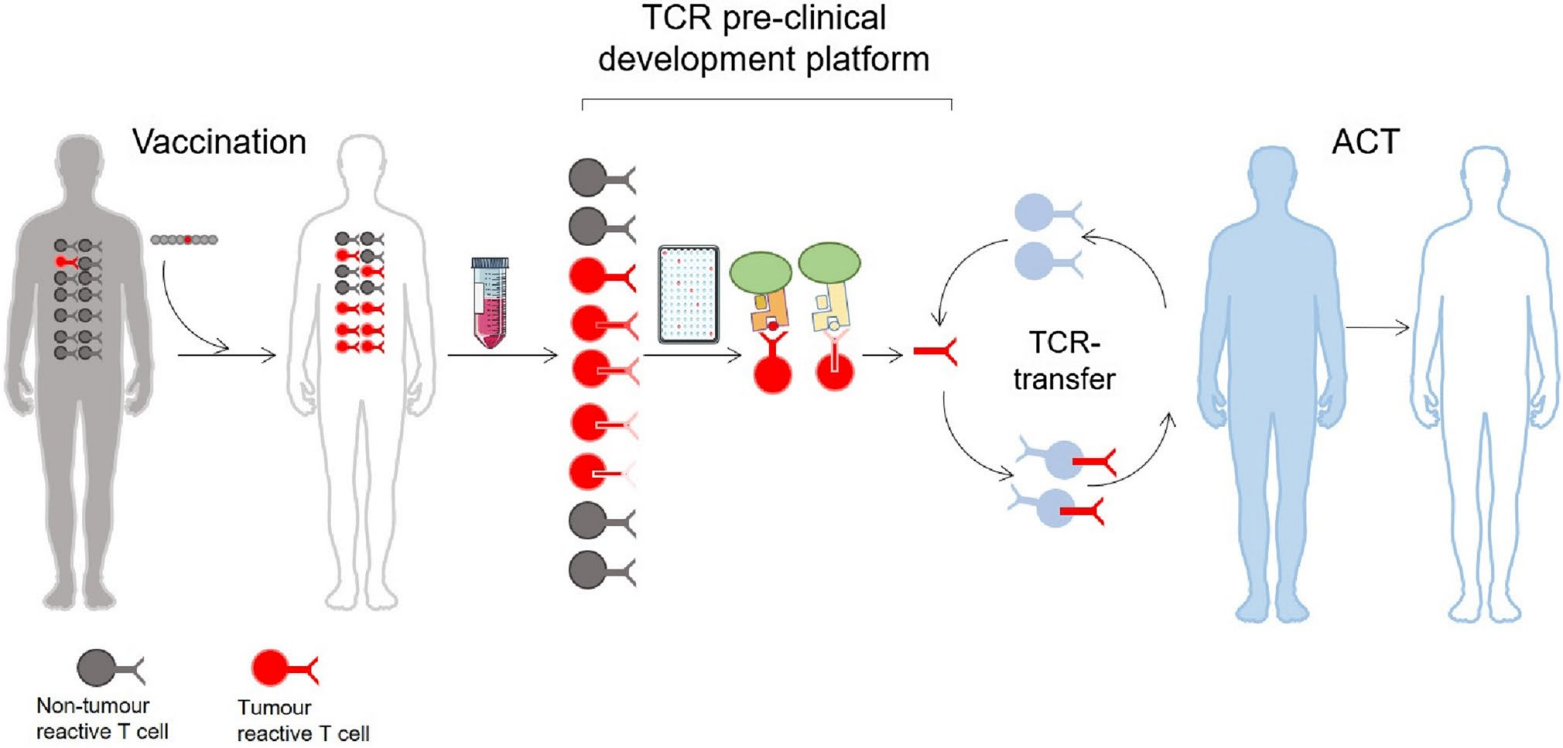
Pre-clinical TCR development platform. Successful vaccination of cancer patients gives rise to an increased frequency of tumour specific T cells. After isolation of the clones and characterization of their pMHC specificity, the TCR sequence is identified. After pre-clinical validation, these therapeutic TCRs are used to treat non-responding MHC-matched patients

The isolation of therapeutic TCRs has been a technical challenge. Firstly, because interesting T cells isolated from the blood or the tumour are never pure, and therefore require selection and expansion. Secondly, although the TCR sequence identification has benefitted from technological advances [7, 8], the validation procedures following the expression and testing of the TCR are still costly and time consuming. As previously mentioned, the signal detected upon TCR stimulation normally gives a weaker cytokine response compared to that of a CAR construct [10]. However, weaker signal observed in vitro does not preclude good clinical efficiency, rather the contrary as exemplified by recent studies with lower affinity [6, 7]. In other words, our in vitro methods of TCR stimulation may lead to an underestimate of their efficacy in vivo. Consequently, this has driven an effort to manufacture supranatural TCRs through affinity maturation [11], murinization [12] or modification of the TCR structure to stabilize it (reviewed in [13]). TCR affinity increased beyond the physiological range comes with the risk of creating novel molecules with unpredictable binding specificities to structurally related peptide sequences from different antigens, creating off-target toxicity [8, 9]. Moreover, TCR variants were tested and the existence of a TCR affinity-related activation threshold was demonstrated both in vitro [14] and in vivo [15]. Here the authors of the two studies proposed that this threshold marked the limit between epitope recognition and autoimmunity. It was further shown that enhancing the peptide affinity, thus modifying the binding threshold on the peptide side, had similar deleterious effect on the in vivo potency of the TCR [16]. Together these reports suggest that TCRs with normal affinity, or unenhanced TCR, might be more optimal and safer for TCR-based therapy. For these reasons, we generally consider the manipulation of TCRs to be too hazardous and unpredictable to be exploited as a living drug. We have therefore implemented a therapeutic TCR platform which only produces “natural” TCRs (Fig. 1). Blood from long-term surviving cancer patients who experienced clinical benefit after treatment with therapeutic cancer vaccines was collected. To find tumour-reactive TCRs, the T cells were isolated and analysed for their pMHC specificity against autologous presenting cells [17–21]. We see three critical advantages of using these TCRs: (1) they were not toxic to the patient who carried them, (2) they were selected in a human thymus, and (3) these TCRs were likely part of the immune response involved in the survival of the patients. Furthermore, these patients can be a source of discovery of TCRs with specificities for alternative antigens; indeed we have previously isolated TCRs targeting tumour antigens other than the vaccine. This suggests that the immune response against these new epitopes was generated by epitope spreading which occurred during the immune attack of the tumour and shown to correlate with clinical response [21]. T-cell clones isolated from these patients can be tested and compared to find the optimal candidates for TCR therapy. The criteria of selection are several, with the most important being (1) a focus on frequently expressed MHC alleles for a broad population coverage, and (2) public rather than personal cancer antigens. Isolating such TCRs and transferring them to new cells will only transfer the properties of the TCR with its specificity and affinity for the target whereas the avidity of the original T cell clone comprising more than the TCR properties will of course not be transferred. The New York esophageal squamous cell carcinoma 1(NY-ESO-1) is by far the most frequently targeted antigen in clinical trials of TCR therapy to date [22]. TCRs specific for this antigen have been tested in several clinical trials ([23–25] and unpublished clinical studies) with variable success. Still, NY-ESO-1 is a perfect target: it is not expressed in normal tissues and is present in different tumour types. In addition, hotspots of antigenic peptides inducing CD4 and CD8 T-cell responses have been well characterized [26, 27]. Recently, the team of D. Baltimore reported on a procedure to isolate the most efficient candidates directly from patient blood [28]. It will be interesting to follow the clinical progression of these TCRs.

We have previously reported on the isolation of a public neoantigen-specific TCR, Radium-1 [29, 30]. Here microsatellite instable (MSI)+ colorectal cancer patients had been vaccinated with a long peptide covering a frequent Transforming growth factor β receptor II (TGFβRII) frameshift mutation as a result of the dysfunctional DNA mismatch repair mechanisms ([31] and Inderberg and Gaudernack et al., unpublished data). From one of these patients, a TCR specific for a known HLA-A*02:01 epitope was isolated [29]. The TCR, named Radium-1, is currently in clinical testing (NCT03431311). We also isolated two MHC class II-restricted TCRs from two other vaccinated patients in this study ([32] and Inderberg, Wälchli et al., unpublished data) which shifted the focus of our development platform to CD4 T-cell derived TCRs due to the central role of CD4 T cells in anti-tumour immune responses.

### The rise of T helper TCRs

**Fig. 2:**
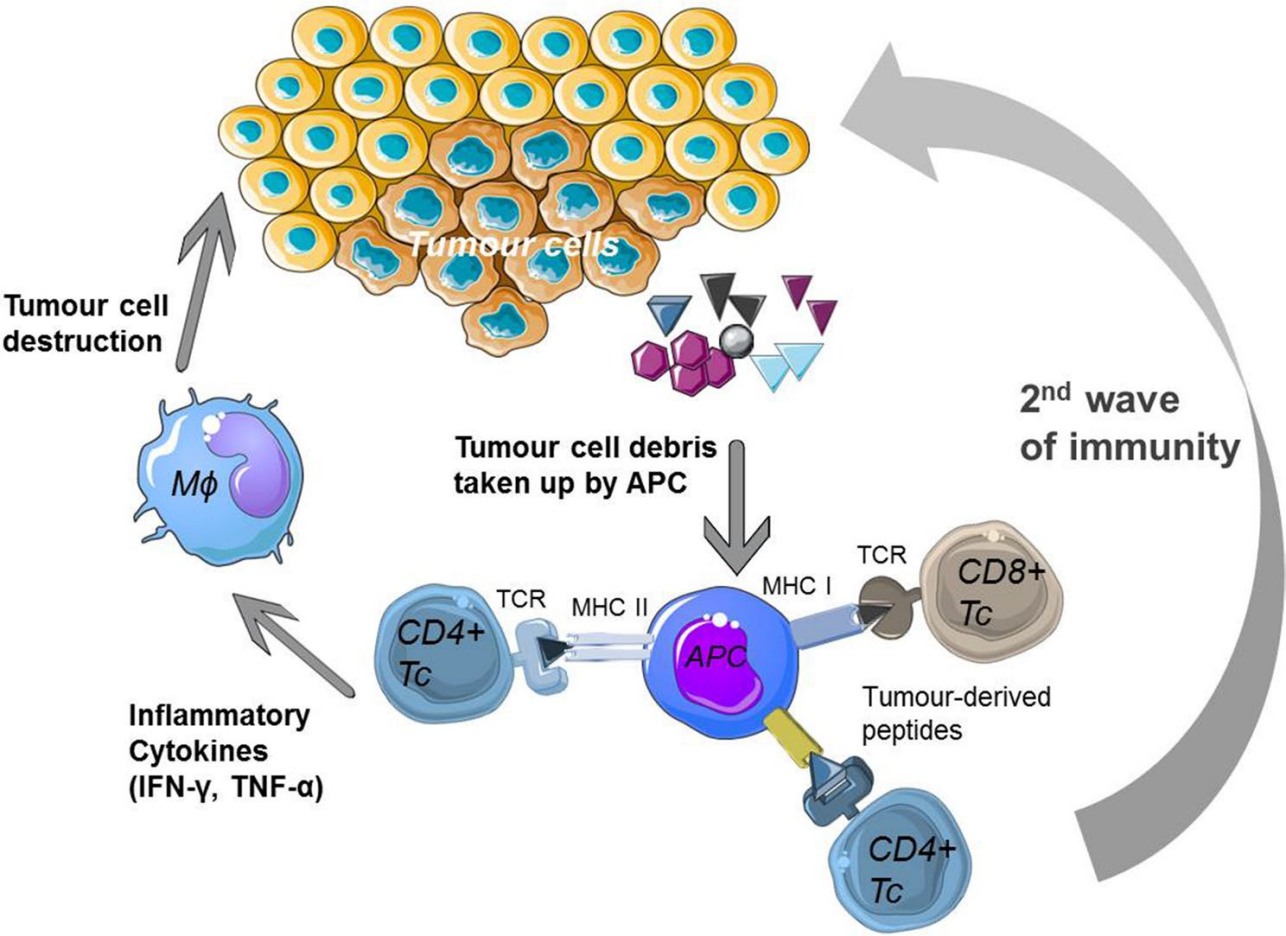
CD4 T-cell anti-tumour responses. Tumour-specific CD4 T cells can activate and maintain CD8 T cell responses and can also induce tumour cell killing by activating tumour-infiltrating macrophages. Graphical elements adapted from Servier Medical Art repository (https://www.servier.com)

Whereas cellular anti-tumour immune responses have typically been attributed to CD8 T cells, CD4 T cells play a critical role in tumour elimination and in the priming and maintenance of CD8 T-cell responses (recently reviewed in [33], Fig. 2). Moreover, CD4 T cells activate innate cells such as macrophages and NK cells to contribute to anti-tumour responses and can also have direct cytotoxic effect against tumour cells expressing MHC class II [34–36]. The use of MHC class II-restricted CD4 T cells for adoptive immunotherapy has been limited due to (1) a lack of well-characterized shared tumour antigens presented by MHC class II, (2) the majority of tumour cells being class II negative and therefore not directly presenting antigen to CD4 T cells, and (3) the lack of tools to evaluate CD4 TCR efficacy. However, the use of CD4 T cells in ACT should also circumvent one of the common tumour escape mechanisms, which is the loss of MHC class I to prevent recognition by the immune system [37]. All clinical studies of TCR therapy published to date, except one [38], have used MHC class I restricted TCRs (recently reviewed in [39]). MHC class II-restricted CD4+ T cells are able to induce more robust and broader anti-tumor immune responses which could improve outcomes in cancer immunotherapy. One of the antigens we have focused on is human telomerase reverse transcriptase (hTERT) with several academic vaccination studies carried out [17–19, 40, 41]. This is a well characterized antigen which is almost universally expressed in cancer cells due to its essential role in unlimited cell growth, metastasis and expression in cancer stem cells (reviewed in [42]). One concern regarding this antigen has been that as an overexpressed tumour-associated antigen (TAA), it is also present at lower level in normal cells such as activated lymphocytes, stem cells and germ cells (reviewed in [42]).

Two MHC class I-restricted TCRs specific for hTERT have previously been published [43, 44]. The HLA-A*02:01 restricted TCR was isolated from a vaccinated HLA transgenic mouse whereas the HLA-A*24:02 restricted TCR was isolated from healthy donor blood. Both were of high affinity and deemed safe by pre-clinical testing, but have not yet reached the clinic. We have, however, not been able to generate tumour-recognizing MHC class I restricted T cell clones against hTERT as the cells may commit fratricide, although there exists conflicting evidence for this [45, 46]. Thus, TAA can become dangerous as target for CD8 TCR due to the risk of “on-target, off-tumour toxicity”. Since only a limited number of normal cells are MHC class II positive, it is tempting to speculate that a CD4 TCR might be less toxic against overexpressed TAA. In agreement with this, we reported that the patients carrying these hTERT-specific T cell clones performed well clinically and had no signs of toxicity. Bone marrow function was indeed monitored in long-term surviving lung cancer patients post vaccination without showing toxicity [18]. In addition, the presence of these hTERT-specific CD4 T cells after vaccination has been shown to correlate with enhanced survival of the patient [19, 20, 40].

We have therefore focused on the development of non-modified patient-derived hTERT-specific TCRs. From a long-term surviving pancreatic cancer patient, we have isolated an hTERT-specific TCR restricted to the very common allele HLA-DP4, which we named Radium-4, which demonstrated surprising qualities (Dillard et al., in revision), such as the capacity to directly eliminate MHC class II positive target cells loaded with peptide, but also to reduce tumour growth in a melanoma xenograft model. The TCR transfected and transduced T cells did not show any signs of fratricide or reactivity against normal cells.

We are also developing additional hTERT-specific TCRs recognising alternative hTERT epitopes after vaccination-induced epitope spreading which are restricted to various HLA alleles [21]. These TCRs are isolated from several patients vaccinated with a hTERT peptide vaccine [21]. Developing CD4 T-cell based therapy comes with the technical challenge that although they can kill tumour cells, their primary mode of action is by engaging with other players in the immune system to orchestrate a broad attack of the tumour. This is impossible to fully reproduce in our in vitro models or in mouse xenograft models using immunodeficient animals. We therefore depend on demonstrating the direct killing effect or cytokine production, which is more circumstantial evidence for their activity, whereas we may be largely underestimating the effect this may have in humans if successfully implemented.

CD4 T-cell derived TCRs are also being developed against other antigens, including neoantigens such as the abovementioned TGFβRII frameshift mutation found in MSI + colorectal and endometrial cancer. Two of the identified MHC class II restricted TCRs were shown to recognise the same mutation as our MHC class I restricted TCR [29, 30].

These TCRs, named Radium-5 and -6, were identified in patients from the same clinical vaccination trial (Inderberg, Gaudernack et al., unpublished data) and shown to efficiently redirect T cells [32]. Preliminary data from our in vivo xenograft model indicate that these TCRs could be as efficient at reducing tumour growth as their MHC class I restricted counterpart (Dillard, Wälchli and Inderberg et al., unpublished observations). The TCRs are restricted to frequently expressed MHC class II molecules and it could be interesting to also combine their use with the Radium-1 TCR.

Finally, the TCRs that we have identified so far do seem to be able to function without their CD8 or CD4 co-receptor. A good illustration was done when we expressed these TCRs in a cell line devoid of co-receptor, such as an NK cell line, the NK-TCR [47]. When we transduced our TCRs into NK92, which had been modified to overexpress human CD3, we observed that Radium-1 TCR was functional in this system, independently of the presence of CD8. Likewise, when we tested some of our CD4 T cell-derived TCRs to generate NK-TCR cells, we observed that these cells killed MHC class II^+^ antigen presenting tumour cells (Mensali, Inderberg and Wälchli et al., unpublished data). These data suggest that vaccinated patient-derived TCR can function without co-receptors.

### Future perspectives

TCR-based therapy also encounters hurdles for eradicating metastatic tumour due to the immunosuppressive tumour microenvironment (TME) or T cell exhaustion. There are several initiatives to remedy this by improving T cell trafficking through the modification of homing receptors [48–50], resistance to TGF-β [51], modification or blocking of immune checkpoints [52]. Such strategies will likely be increasingly implemented in future clinical trials. Additionally, there are numerous therapy combinations that, if designed wisely, could have an impact on the efficacy of ACT, such as the combination with immune checkpoint blockade, post-transplant vaccination to improve T-cell persistence, and specific targeting of the TME immunosuppression. To date, few CD4 T-cell based therapies have been tested clinically [38, 53–55], but have shown clear evidence of clinical activity.

Combining HLA class I- and class II-restricted TCRs for T-cell redirection may also provide a more potent therapeutic effect in adoptive T cell therapy [56]. MHC class II-restricted TCRs may additionally have direct therapeutic value both in haematopoietic malignancies and in melanoma where tumour cells frequently express MHC class II. Importantly, CD4 T cells and MHC class II TCRs therefore have the potential to orchestrate broad and long-lasting immune responses that enable cancer control.

## Abbreviations

ACT: Adoptive cell therapy
CAR: Chimeric antigen receptor
hTERT: Human telomerase reverse transcriptase
MSI: Microsatellite instability
NY-ESO-1: New York esophageal squamous cell carcinoma 1
pMHC: Peptide MHC
TCR: T cell receptor
TGFβRII: Transforming growth factor β receptor II
